# Supplementary material for: Spatiotemporal dynamics and influencing factors of soil heterotrophic respiration in northeast China
Source: PLoS One. 2025 May 27;20(5):e0322920. doi: 10.1371/journal.pone.0322920 (PMC12112202; doi:10.1371/journal.pone.0322920)
Supplement: Attachment — Submitted filename: Response to Reviewers.docx [file pone.0322920.s003.docx]

Dear Dr. Prasath,

Thank you very much for the thoughtful and constructive feedback on our manuscript entitled "Spatiotemporal dynamics and influencing factors of soil heterotrophic respiration in Northeast China." We are truly grateful for the time and effort you and the reviewers have invested in evaluating our work. Your detailed comments and suggestions have significantly improved the clarity and quality of our manuscript.

We have carefully addressed each of the reviewers' comments and made the necessary revisions to the manuscript:

Please include your amended statements within your cover letter; we will change the online submission form on your behalf.

**Response:** We have made revisions and provided explanations for each of the comments.

1. We note that Figures 4,5,6 and 7 in your submission contain [map/satellite] images which may be copyrighted. All PLOS content is published under the Creative Commons Attribution License (CC BY 4.0), which means that the manuscript, images, and Supporting Information files will be freely available online, and any third party is permitted to access, download, copy, distribute, and use these materials in any way, even commercially, with proper attribution. For these reasons, we cannot publish previously copyrighted maps or satellite images created using proprietary data, such as Google software (Google Maps, Street View, and Earth). For more information, see our copyright guidelines: http://journals.plos.org/plosone/s/licenses-and-copyright.

**Response:** The data is sourced from vector data provided by National Catalogue Servic for Geographic Information. The URL to obtain the data is https://www.webmap.cn/commres.do?method=result100W. The database is named "1:1,000,000 Scale Public Basic Geographic Information Data (2021)". The data can be obtained for free.

(Lines 155-156)

3. Please remove your figures from within your manuscript file, leaving only the individual TIFF/EPS image files, uploaded separately. These will be automatically included in the reviewers’ PDF.

**Response:** The figures in the manuscript have been removed.

Reviewer #1: General Comments:

1. English Language: The manuscript requires a thorough check for grammar and language improvements.

**Response: We have checked for the grammar and language improvements.**

2. Citation Pattern: Ensure the citation style is consistent throughout the manuscript.

**Response:** We have checked the citation style and ensured consistency throughout the manuscript.

Specific Comments:

1. Line 40: Correct citation format needed.

**Response:** The citation 'Le et al. 2014' has been modified to use superscript numbers for referencing.

2. Line 54: Clarify whether this refers to laboratory calibration.

**Response:** The term "taboratory calibration" was a typographical error, and it should be corrected to "laboratory calibration."

(Lines 54-56)

3. Lines 66 & 67: Avoid repetition; rewrite the sentence for clarity.

**Response:** we have revised the sentence to improve clarity and remove redundancy. The revised text now presents the information in a more concise and straightforward manner.

(Lines 66-67)

4. Line 71: Expand the term "Ra" when mentioned for the first time.

**Response:** we have expanded the term 'Ra' (Autotrophic Respiration) the first time it is mentioned in the manuscript.

(Line 72)

5. Line 73: Provide a reference as it is mentioned as "sensitive zone."

**Response:** we have added relevant references to support the statement that 'Northeast China is located in the sensitive zone of global climate change.' Specifically, we have included the following references that discuss the climate sensitivity and vegetation dynamics in Northeast China:

Li H., Zhang H., Li Q., Zhao J., Guo X., Ying H., et al. (2021). Vegetation productivity dynamics in response to climate change and human activities under different topography and land cover in Northeast China. *Remote Sensing, 13*(5), 975. [doi:10.3390/rs13050975](https://doi.org/10.3390/rs13050975).

Li C., Chen Y., Huang Y., Zhang L., Wu S., Zhang W., et al. (2022). Sensitivity Analysis of Precipitation Pattern as Indicator of Climatic Warming in High Latitudes of Northeast China. *Sensors and Materials, 34*, 4263. [doi:10.18494/SAM3984](https://doi.org/10.18494/SAM3984).

(Line 75)

6. Materials and Methods Section: Include a map marking all relevant regions.

**Response:** We have added a map of the study area in the Materials and Methods section (figure 1), including the location of the study area, as well as the annual average temperature and precipitation within the study area.

7. Lines 105 & 106: Rewrite the sentence for clarity and flow.

**Response:** We have revised the 'Land cover type' section to improve the clarity and flow of the language.

(Lines 109-116)

8. Line 155: Ensure citation adheres to journal instructions.

**Response:** The citation format has been revised to ensure it complies with the journal's requirements.

(Line 163)

9. Lines 172 & 173: Clarify or rewrite the statement about soil respiration values.

**Response:** We have reorganized the language and revised this paragraph to make the statement about soil respiration values clearer and more logical.

(Lines 221-250)

10. Lines 186-190: Justify the inclusion of this section in "Materials and Methods" and mention any statistical analyses performed.

**Response:** In this study, we applied certain environmental constraints within the GSMSR model to reflect realistic ecological conditions in Northeast China. Specifically, the soil respiration rate is assumed to remain constant when the soil organic carbon density is greater than or equal to 15.09 kg/m² at the 20 cm soil depth, as this value represents a threshold commonly observed in the literature for soil organic matter content influencing respiration rates. Additionally, we set the soil respiration rate to zero at temperatures below -15.96℃ and at its maximum when temperatures exceed 30.96℃, which is based on observed physiological responses of soil microbial activity to temperature extremes. These constraints were included to ensure the accuracy and ecological realism of the model, based on field data and environmental conditions typical for the region.

No statistical analysis was performed specifically for the derivation of these thresholds. They were selected based on literature values and expert judgment in the field ^[1, 2]^. Future work may involve statistical validation of these thresholds using local data.

We have added references in the manuscript.

(Lines 210-214)

11. Line 195: Correct spelling errors.

**Response:** The phrase 'he proportion' has been corrected to 'heproportion'.

(Line 219)

12. Lines 215 & 216: Justify the claim regarding minimal differences between grassland, farmland, and forest results with statistical analysis.

**Response:** We conducted a non-parametric independent sample test to verify the statistical significance of the differences in annual mean soil respiration and carbon release across the various land use types.

The results of the test indicated that there are significant differences (P < 0.001) in the annual mean soil respiration and carbon release among the land use types. This provides statistical support for the differences observed between grassland, farmland, and forest, and further strengthens the conclusions drawn in our study.

(Lines 229-232)

13. Line 244: Rewrite the sentence for better clarity.

**Response:** We have revised the sentence for better clarity and flow, as suggested. The revised version now clearly conveys the spatial distribution trends of different ecosystems and the axial ratio of Rh in various land types.

(Lines 268-271)

14. Lines 322, 329, 330, 331: Check for spelling errors.

**Response:** The spelling errors have been corrected.

(Lines 317, 417, 418, 419)

15. Lines 364 & 365: Rewrite for better clarity.

**Response:** We have revised the sentence for better clarity and flow, as suggested.

(Lines 365-367)

16. Lines 383 & 384: Check for spelling errors and ensure proper citation as per journal instructions.

**Response:** We have carefully checked the spelling and made necessary corrections. Additionally, we have ensured that the citations are formatted according to the journal's guidelines.

(Lines 382-384)

Reviewer #2: The manuscript entitled "Spatiotemporal dynamics and influencing factors of soil heterotrophic respiration in Northeast China" explores the spatiotemporal variations of soil heterotrophic respiration (Rh) in Northeast China from 2001 to 2020. It employs the GSMSR model, along with advanced spatial analysis techniques, to identify patterns and determine influencing factors, providing insights into regional carbon flux dynamics.

The manuscript is comprehensive, addressing a critical topic in soil science and carbon cycle research. It effectively integrates modeling, spatial analysis, and ecological insights. However, improvements in language clarity and the addition of more detailed methodological descriptions could enhance its academic quality.

General Comments:

1. Ensure consistency in units and terminology throughout the manuscript (e.g., kgC/ha/year vs kgC·ha⁻¹·yr⁻¹).

**Response:** The units have been standardized to kgC/ha/year.

2. Revise the introduction to better align with the objectives and hypotheses.

**Response:** I have clearly outlined the challenges related to the lack of large-scale Rs measurement methods and data, which directly led to the formulation of the research objectives. Additionally, I have explicitly stated the hypotheses in relation to spatial variations in Rs and the impact of regional differences in vegetation coverage and economic development. These changes aim to ensure a smoother connection between the introduction and the core research questions. Additionally, I have also revised the language expression in the introduction to improve its clarity, fluency, and conciseness.

3. Clarify the novelty of this research compared to similar studies using the GSMSR model.

**Response:** We have included a brief explanation of the strengths of the GSMSR model compared to other models in the revised manuscript. Specifically, we highlight that although the GSMSR model requires substantial spatial data and has higher computational demands, its geostatistical framework allows it to effectively capture the spatial heterogeneity of soil respiration. This ability makes it particularly suitable for complex geographical and climatic conditions, and for long-term time-series data analysis. Compared to process models like BIOME-BGC and DAYCENT, which have more complex parameterization, the GSMSR model offers a simpler parameter framework, while still capturing the spatial variations and ecological complexities of soil respiration more effectively than simplified models such as CASA and Q10. Additionally, unlike machine learning models, GSMSR does not rely on large training datasets and provides better interpretability, making it an ideal choice for our study.

4. Include more robust discussions on potential uncertainties in the model results.

**Response:** As mentioned, while assuming constant SOC simplifies the soil respiration dynamics, this assumption fails to capture the fluctuations in the SOC mineralized substrate and the changes in its temperature sensitivity. These oversights may lead to biases in long-term predictions and the model's response to climate change. Additionally, it may hinder accurate estimations of the effects of microbial community composition and nutrient cycling on soil respiration (Rh). We revise the manuscript to include discussion of these potential errors and their implications for model accuracy.

5. Proofread the manuscript to address grammatical errors and enhance readability.

**Response:** We have checked for the grammar and language improvements.

Line-to-Line Specific Comments:

1. Line 19: Revise "Soil heterotrophic respiration (Rh) is the main way of carbon output" to "Soil heterotrophic respiration (Rh) represents a primary pathway of carbon release from soil."

**Response:** Already revised.

(Line 19)

2. Line 27: Clarify the units used for carbon release ("4.76×10¹¹ kg/year") to ensure interpretability.

**Response:** We have revised the sentence to clarify the units used for carbon release. The units are now explicitly stated as "kilograms per year (kg/year)", ensuring better interpretability of the data. We have also added a brief explanation that these values represent the annual carbon flux in the study region.

(Line 29)

3. Line 30: Expand on the term "standard deviation ellipse" for readers unfamiliar with spatial statistics.

**Response:** Added “a statistical method that describes the spread and direction of data points in space” to briefly explain the standard deviation ellipse.

(Line 24-25)

4. Line 62: Replace "calculate" with "calculated" for grammatical accuracy.

**Response:** By changing 'Rh was calculated and its spatio-temporal variability was analyzed based on process models.' to 'Calculating Rh and analyzing its spatio-temporal variability based on process models,' the grammatical accuracy and fluency have been ensured.

(Lines 63-64)

5. Line 73: Consider adding more context to explain why Northeast China is a "sensitive zone of global climate change."

**Response:** Two references^[3, 4]^ have been added to support the statement 'Northeast China is a sensitive zone of global climate change.'

(Line 75)

6. Line 123: Ensure mathematical symbols in formulas are consistent with conventions (e.g., subscripts).

**Response:** According to international conventions, O is modified to $\overline{O}$ to represent the average of the measured value

(Lines 129-131)

7. Line 148: Provide the reasoning for selecting 500m resolution for DEM data.

**Response:** We resampled the 250m resolution DEM data to a 500m spatial resolution for consistency with other datasets used in this study. The MCD12Q1 land cover dataset, which is one of the primary data sources for this research, has a spatial resolution of 500m. Additionally, the soil organic carbon density data used for model calibration has a coarser resolution of 1km. By resampling the DEM data to 500m, we ensured consistency in the spatial resolution across all datasets, which is important for accurate spatial analysis and model integration. This resolution was also selected for its balance between data detail and computational efficiency.

In the original text, it was specifically modified to “We downloaded the 250m resolution data covering the study area and resampled it to 500m for consistency with other datasets used in this study, such as the MCD12Q1 land cover dataset with 500m resolution, and the soil organic carbon density data with 1km resolution. This resampling ensured uniformity in spatial resolution across all datasets, which is important for accurate spatial analysis and model integration.”

(Lines 149-154)

8. Line 163: Include a brief explanation of the GSMSR model's strengths compared to other models.

**Response:** We have included a brief explanation of the strengths of the GSMSR model compared to other models in the revised manuscript. Specifically, we highlight that although the GSMSR model requires substantial spatial data and has higher computational demands, its geostatistical framework allows it to effectively capture the spatial heterogeneity of soil respiration. This ability makes it particularly suitable for complex geographical and climatic conditions, and for long-term time-series data analysis. Compared to process models like BIOME-BGC and DAYCENT, which have more complex parameterization, the GSMSR model offers a simpler parameter framework, while still capturing the spatial variations and ecological complexities of soil respiration more effectively than simplified models such as CASA and Q10. Additionally, unlike machine learning models, GSMSR does not rely on large training datasets and provides better interpretability, making it an ideal choice for our study.

(Lines 169-178)

9. Line 171: Expand on the choice of static chamber/GC method for soil respiration measurement.

**Response:** We have expanded on the choice of the static chamber/GC method in our manuscript. We have added that research has shown both the static chamber/GC method and the static/dynamic chamber/IRGA methods provide reliable localized soil respiration data across different soil types and climatic conditions ^[5, 6]^. This addition helps clarify the reliability and applicability of these methods for soil respiration measurements in various environments."

(Lines 187-189)

10. Line 196: Justify the use of quadratic curve fitting for calculating Rh.

**Response:** Soil respiration rates are influenced by seasonal variations and typically exhibit a nonlinear pattern. The quadratic curve fitting effectively captures this variation. We also performed a goodness-of-fit test, with an R² value of 0.5922, indicating that the fitting results are highly accurate. Therefore, we believe that the use of quadratic curve fitting is reasonable and provides reliable results for the study.

11. Line 207: Explain why farmland has the highest Rh values among land use types.

**Response:** High soil organic carbon input (crop residues, fertilization), frequent soil disturbances (tillage) that increase aeration and microbial activity, and stable soil moisture conditions that are favorable for microbial growth—these combined factors contribute to the highest heterotrophic respiration in agricultural soils among all land use types.

(Lines 236-240)

12. Line 227: Clarify the significance of azimuth change range values in spatial analysis.

**Response:** The azimuth angle can be used to identify the spatial aggregation characteristics of Rh.

(Line 255)

13. Line 271: Include ecological reasons for cold and hot spots observed in different regions.

**Response:** The hotspot areas have soil rich in organic matter, coupled with frequent soil disturbances and suitable soil moisture and temperature conditions, making them hotspots for Rh. In contrast, the cold spot areas are mainly characterized by plains and plateaus, with grassland and farmland ecosystems, where the relatively low soil organic matter content is likely the main reason.

(Lines 284-286)

14. Line 307: Justify excluding meteorological factors in the analysis of influencing factors.

**Response:** The GSMSR model has already incorporated temperature and precipitation as primary drivers to simulate soil heterotrophic respiration (Rh), meaning that the effects of these meteorological factors are implicitly captured in the model's outputs. Consequently, we focus on factors that may independently influence Rh beyond the model’s driving variables. We hypothesize that vegetation cover and the level of economic and social development could have significant impacts on Rh, aspects that may not have been sufficiently explored in previous studies. By examining these factors, we aim to provide a more comprehensive understanding of the diverse influences on Rh.

(Lines 308-314)

15. Line 327: Provide references to support the claim that high vegetation coverage decreases soil aeration.

**Response:** Tang^[7]^ and Raich^[8]^ support the idea that high vegetation cover reduces soil aeration.

(Line 322)

16. Line 339: Explain how local economic activities directly influence Rh rates with specific examples.

**Response:** We obtained data from the China National Data Network (<https://data.stats.gov.cn/>), which shows that the area of crop cultivation, the use of organic fertilizers, and GDP have all increased. This active agricultural production provides a continuously increasing carbon source for microbial communities, thereby promoting an increase in Rh.

(Lines 333-336)

17. Line 350: Discuss potential errors introduced by assuming constant SOC in the model.

**Response:** As mentioned, while assuming constant SOC simplifies the soil respiration dynamics, this assumption fails to capture the fluctuations in the SOC mineralized substrate and the changes in its temperature sensitivity. These oversights may lead to biases in long-term predictions and the model's response to climate change. Additionally, it may hinder accurate estimations of the effects of microbial community composition and nutrient cycling on soil respiration (Rh). We revise the manuscript to include discussion of these potential errors and their implications for model accuracy.

(Lines 357-364)

18. Line 370: Include a definition for NPP (Net Primary Productivity) for clarity.

**Response:** We have added a definition for Net Primary Productivity (NPP) in the manuscript. Specifically, we defined NPP as the amount of carbon assimilated by plants through photosynthesis minus the carbon released through plant respiration.

(Lines 369-372)

19. Line 381: Expand on how land use optimization could mitigate Rh variability.

**Response:** We have expanded on how land use optimization could mitigate Rh variability. We have highlighted that by promoting sustainable agricultural and forestry practices, such as reduced tillage, agroforestry, and rotational cropping, soil organic carbon levels can be maintained or increased, which helps stabilize Rh. Additionally, incorporating organic farming techniques, such as crop residue return and cover crops, can boost vegetation coverage and improve the soil’s carbon sequestration potential, thus reducing Rh fluctuations. Furthermore, optimizing irrigation and fertilization practices can help maintain soil moisture and nutrient balance, further reducing Rh variability. These strategies can enhance the resilience of ecosystems to climate change and buffer against extreme events, ultimately mitigating Rh variability across regions.

(Lines 416-426)

20. Line 403: Provide examples of human management modes affecting Rh.

**Response:** we have expanded the manuscript to include specific examples of human management practices that affect Rh. We highlighted how intensive fertilization, pesticide use, irrigation practices, and straw returning can influence soil nutrient levels, microbial activity, and organic matter decomposition, all of which impact Rh. These examples help clarify how different human management modes can significantly affect Rh and its sensitivity to vegetation coverage changes in farmland ecosystems.

(Lines 418-424)

21. Line 410: Suggest practical recommendations for improving vegetation coverage in degraded regions.

**Response:** we have added practical recommendations for improving vegetation coverage in degraded regions. We emphasize land use optimization as a key strategy, promoting more sustainable agricultural and forestry practices that enhance soil organic matter content and microbial activity. Specific practices such as reduced tillage, agroforestry, rotational cropping, and the incorporation of organic farming techniques like crop residue return and cover crops are highlighted. These methods can help increase vegetation coverage and improve the carbon sequestration potential of soils, thus reducing Rh fluctuations. We believe these recommendations provide actionable steps for improving vegetation coverage in degraded areas, contributing to the restoration of ecosystems and enhancing their carbon sequestration capacity.

(Lines 416-426)

22. Figure 4 Legend: Improve the legend to ensure accessibility for readers unfamiliar with GIS methods.

**Response:** The figure (originally Figure 4) has been redrawn, and the legend now explains the Center of the Ellipse and the Spatial Distribution Ellipse.

23. Table 2: Highlight key findings or trends to guide readers in interpreting the data.

**Response: We** have highlighted the key findings in the revised section, emphasizing the small differences between the simulated and measured values across different ecosystems. This highlights the GSMSR model’s accuracy in predicting soil respiration rates, particularly in wetland and forest ecosystems. These findings help guide readers in interpreting the data and underscore the model's applicability to various land use types.

(Lines 210-214)

**Reference**

1.	Yu G, Zheng Z, Wang Q, Fu Y, Zhuang J, Sun X, et al. Spatiotemporal Pattern of Soil Respiration of Terrestrial Ecosystems in China: The Development of a Geostatistical Model and Its Simulation. Environmental Science & Technology. 2010;44(16):6074-80. doi: 10.1021/es100979s.

2.	Bond-Lamberty B, Thomson A. Temperature-associated increases in the global soil respiration record. Nature. 2010;464(7288):579-82. doi: <https://doi.org/10.1038/nature08930>.

3.	Li H, Zhang H, Li Q, Zhao J, Guo X, Ying H, et al. Vegetation Productivity Dynamics in Response to Climate Change and Human Activities under Different Topography and Land Cover in Northeast China. Remote Sensing. 2021;13(5):975. doi: <https://doi.org/10.3390/rs13050975>. PubMed PMID: doi:10.3390/rs13050975.

4.	Li C, Chen Y, Huang Y, Zhang L, Wu S, Zhang W, et al. Sensitivity Analysis of Precipitation Pattern as Indicatorof Climatic Warming in High Latitudes of Northeast China. Sensors and Materials. 2022;34:4263. doi: <https://doi.org/10.18494/SAM3984>.

5.	Yim MH, Joo SJ, Nakane K. Comparison of field methods for measuring soil respiration: a static alkali absorption method and two dynamic closed chamber methods. Forest Ecology and Management. 2002;170(1):189-97. doi: <https://doi.org/10.1016/S0378-1127(01)00773-3>.

6.	Heinemeyer A, McNamara NP. Comparing the closed static versus the closed dynamic chamber flux methodology: Implications for soil respiration studies. Plant and Soil. 2011;346(1):145-51. doi: <https://doi.org/10.1007/s11104-011-0804-0>.

7.	Tang X, Fan S, Du M, Zhang W, Gao S, Liu S, et al. Spatial and temporal patterns of global soil heterotrophic respiration in terrestrial ecosystems. Earth Syst Sci Data. 2020;12(2):1037-51. doi: 10.5194/essd-12-1037-2020.

8.	Raich JW, Schlesinger WH. The global carbon dioxide flux in soil respiration and its relationship to vegetation and climate. Tellus B. 1992;44(2):81-99. doi: <https://doi.org/10.1034/j.1600-0889.1992.t01-1-00001.x>.
